# Crystal Structure of an Intramolecular Mesaconyl-Coenzyme A Transferase From the 3-Hydroxypropionic Acid Cycle of *Roseiflexus castenholzii*

**DOI:** 10.3389/fmicb.2022.923367

**Published:** 2022-05-26

**Authors:** Zhenzhen Min, Xin Zhang, Wenping Wu, Yueyong Xin, Menghua Liu, Kangle Wang, Xingwei Zhang, Yun He, Chengpeng Fan, Zhiguo Wang, Xiaoling Xu

**Affiliations:** ^1^Department of Biochemistry and Molecular Biology, School of Basic Medical Sciences, The Affiliated Hospital of Hangzhou Normal University, Hangzhou, China; ^2^Photosynthesis Research Center, College of Life and Environmental Sciences, Hangzhou Normal University, Hangzhou, China; ^3^Department of Biochemistry and Molecular Biology, School of Basic Medical Sciences, Wuhan University, Wuhan, China; ^4^Key Laboratory of Aging and Cancer Biology of Zhejiang Province, Hangzhou Normal University, Hangzhou, China

**Keywords:** filamentous anoxygenic phototrophs, 3-hydroxypropionic acid cycle, Mesaconyl-CoA transferase, *Roseiflexus castenholzii*, crystal structure

## Abstract

Coenzyme A (CoA) transferases catalyze reversible transfer of CoA groups from CoA-thioesters to free acids, playing important roles in the metabolism of carboxylic acids in all organisms. An intramolecular CoA transferase, Mesaconyl-CoA C1-C4 CoA transferase (MCT) was identified in the autotrophic CO_2_ fixation pathway, 3-hydroxypropionic acid cycle of filamentous anoxygenic phototrophs (FAPs). Different from the well-known CoA transferases that catalyze CoA transfer between two distinct substrates, MCT specifically catalyzes the reversible transformation of mesaconyl-C1-CoA to mesaconyl-C4-CoA, a key reaction intermediate for carbon fixation. However, the molecular mechanism of MCT in employing one substrate is enigmatic. Here we determined the crystal structure of MCT from a chlorosome-less FAP *Roseiflexus castenholzii* at 2.5 Å resolution, and characterized the catalytic mechanisms through structural analyses and molecular dynamic simulations. The structure of *R. castenholzii* MCT consists of a Rossmann fold larger domain and a small domain that are connected by two linkers. Two MCT subunits are cross interlocked at the linker regions to form a functional dimer in solution, in which the substrate binding pockets are located at the interface of the Rossmann fold larger domain from one subunit and the small domain from the other subunit. In the simulated binding structures, both the substrate mesaconyl-C1-CoA and product mesaconyl-C4-CoA form extensive electrostatic and hydrogen bonding interactions with MCT. But some differences exist in the binding mode of these two CoA analogs, Arg314’ from the second subunit of the dimer presenting dramatic conformational changes in binding with mesaconyl-C4-CoA. Together with Arg47 and one water molecule, a strictly conserved residue Asp165 are essential for catalyzing the reversible intramolecular CoA transfer reaction, through the electrostatic and hydrogen bonding interactions with the mesaconic tail of both the substrate and product. This study revealed a previously unrecognized mechanism for the uncommon intramolecular CoA transfer reaction, which will not only broaden the knowledge on the catalytic mechanisms of CoA transferases, but also contribute to enzyme engineering or biosynthetic applications of the 3-HP cycle for synthesis of fine chemicals and important metabolites.

## Introduction

Coenzyme A (CoA)-transferases catalyze the reversible transfer of CoA groups from CoA-thioesters to free acids, playing essential roles in the metabolism of carboxylic acids in all organisms. Based on the reaction mechanisms and amino acid sequences, these enzymes are grouped into three types. The reaction mechanisms of type I and II CoA transferases both involve the alternate formation of thioester and anhydride intermediates, but their catalytic mechanisms are quite different (Heider, 2001). The type-I enzymes catalyze CoA transfer using a ping-pong mechanism that requires formation of the CoA-thioester intermediate with a glutamate residue of the enzyme, then the enzyme-CoA intermediate reacts with a CoA-acceptor to proceed the reaction. The type-II CoA transferase usually catalyzes a partial reaction of the citrate or citramalate lyases that are comprised of a CoA-transferase (α-subunit), a lyase (β-subunit) and an acyl carrier protein (ACP, γ-subunit) (Buckel and Bobi, 1976; Subramanian et al., 1983). The catalytic mechanism involves ternary-complex formation of the enzyme, donor ACP-thioester (acetyl-ACP) and a thioester-acceptor (citrate or citramalate), without formation of covalently bound intermediates at the enzyme (Buckel et al., 1971; Buckel and Bobi, 1976; Malinin, 1977). Upon conversion of the thiol group of ACP to an acetyl-thioester, the CoA-transferase subunit catalyzes the exchange of free citrate or citramalate against the acetyl group of ACP (Dimroth and Eggerer, 1975; Buckel and Bobi, 1976; Mandal et al., 2000). Similarly, reaction of the type III CoA transferase also requires formation of a ternary complex between the enzyme and two substrates, but these enzymes employ diffusible CoA-thioester instead of ACP-thioester as substrates (Baetz and Allison, 1990; Leutwein and Heider, 1999; Dickert et al., 2000; Elssner et al., 2001; Heider, 2001; Jonsson et al., 2004; Friedmann et al., 2006).

In addition to the above-mentioned CoA transferases that catalyze CoA transfer between two distinct substrates, an intramolecular CoA transferase, Mesaconyl-CoA C1-C4 CoA transferase (MCT) was identified in the autotrophic CO_2_ fixation pathway of a phototrophic bacterium *Chloroflexus aurantiacus* (Zarzycki et al., 2009). As the representative species of filamentous anoxygenic phototrophs (FAPs), *C. aurantiacus* grow heterotrophically in the dark by aerobic respiration but autotrophically in the light through photosynthesis (Pierson and Castenholz, 1974; Strauss and Fuchs, 1993). Instead of Calvin cycle, *C. aurantiacus* and many other FAPs fix carbon through a new metabolic pathway, the 3-hydroxypropionate (3-HP) cycle (Strauss and Fuchs, 1993). This cycle utilizes bicarbonate instead of CO_2_ as substrate, it also assimilates small organic fermentation products even under anaerobic conditions. Most importantly, the 3-HP cycle is coupled by two cycles that ensure efficient utilization of the substrates and reaction intermediates. The first cycle catalyzes acetyl-CoA and two molecules of bicarbonate into one molecule of *(S)*-malyl-CoA, which is then cleaved to glyoxylate and acetyl-CoA. The second cycle starts from condensation of glyoxylate and propionyl-CoA to a *(2R, 3S)*-β-methylmalyl-CoA, and ends with cleavage of *(S)*-citramalyl-CoA into pyruvate and acetyl-CoA. As a result, the bicycle absorbs three molecules of bicarbonate and produces one molecule of pyruvate, with consumption of 5 molecules of ATP and 6 molecules of NADPH (Strauss and Fuchs, 1993; Zarzycki et al., 2009).

In the second cycle, conversion of *(2R, 3S)*-β-methylmalyl-CoA to *(S)*-citramalyl-CoA requires transfer of the CoA moiety from the C1 to C4 carboxyl group of the C5-dicarboxylic acid. However, neither free carboxylic acid nor free mesaconate exchange with mesaconyl-CoA was observed in the reaction. Zarzycki et al. (2009) identified and characterized a new CoA transferase that does not use acetyl-CoA or succinyl-CoA as CoA donors, nor free mesaconate, itaconate, (*R*)-/(*S*)-malate and (*R*)-/(*S*)-citrate as CoA receptors. Instead, it specifically catalyzes the intramolecular transformation of mesaconyl-C1-CoA to mesaconyl-C4-CoA, and therefore named as MCT. Sequence alignment with those of the type III CoA transferases revealed a strictly conserved aspartate residue of MCT, which seems to be capable of forming an acid anhydride with the CoA-activated acid. Reaction of this anhydride with the inhibitor compounds resulted in inactivation of the enzyme. However, MCT was partially inactivated by hydroxylamine that forms a hydroxamate at the CoA-activated glutamate residue of type I CoA transferases (White and Jencks, 1976; Mack and Buckel, 1995; Selmer and Buckel, 1999). Additionally, the enzymatic activity of MCT was nearly not affected by sodium borohydride that reduces the glutamyl-CoA (Mack and Buckel, 1995; Zarzycki et al., 2009), indicating that the active site conformation and catalytic mechanism of MCT are probably different from other CoA transferases. Especially, in comparison with the enzymes that transfer CoA between two different substrates, the molecular mechanism of MCT in catalyzing the intramolecular CoA transfer of only one substrate is enigmatic.

Here, we report the crystal structure of MCT from *Roseiflexus castenholzii*, a chlorosome-less FAP that is closely related to *C. aurantiacus*, wherein MCT was first identified and characterized (Hanada et al., 2002; Zarzycki et al., 2009). Although no substrate or analog has been detected in the structure, the binding modes of CoA, mesaconyl-C1-CoA and mesaconyl-C4-CoA were obtained through structural superimpositions and molecular dynamics (MD) simulations. Based on the crystal structure of apo-MCT and the characterized binding features of these CoA analogs, we proposed a molecular mechanism of *R. castenholzii* MCT in catalyzing the intramolecular CoA-transfer reaction. The results of this study provide structural basis for understanding the catalytic mechanism of intramolecular CoA transfer reactions, it will contribute to the diversity and molecular evolutions of CoA transferases, and their applications in synthesis of 3-HP and other important fine chemicals.

## Materials and Methods

### Protein Expression and Purification

The gene sequence encoding Mesaconyl-CoA transferase from *R. castenholzii* strain DSM 13941 (NC_009767) was inserted into pEASY-E1 expression vector at *Nhe*I and *Sac*I restriction site to construct a N-terminal 6 × His-tagged recombinant expression vector. After DNA sequencing, the plasmid with the correct insert was transformed into *Escherichia coli* BL21 (DE3) cells, the cells were grown in 1 L Luria-Bertani broth containing 100 mg mL^–1^ Ampicillin at 37°C until the OD_600_ reached 0.6–0.8. The gene expression was then induced with 0.1 mM isopropyl-β-D-thiogalactopyranoside (IPTG) overnight at 25°C.

Cells were harvested by centrifugation at 6,000 r.p.m. for 15 min at 4°C, and resuspended in 40 mL wash buffer containing 25 mM Tris–HCl pH 8.0, 300 mM NaCl, 10 mM imidazole, 0.01% Triton X-100 and 0.1 mM phenylmethylsulfonylfluoride (PMSF) prior to the homogenization with a high-pressure homogenizer (Union, People’s Republic of China). The insoluble cell debris was removed by centrifugation at 15,000 rpm for 40 min at 4°C. The supernatant containing crude soluble proteins was loaded onto a Ni^2+^-chelating affinity chromatography column (GE Healthcare, United States) and was rinsed with 100 mL binding buffer (25 mM Tris–HCl pH 8.0, 300 mM NaCl, 20–50 mM imidazole) to remove non-specifically bound proteins. The bound MCT protein was eluted with the binding buffer containing 200 mM imidazole. The eluates were further purified by a HiLoad 16/600 Superdex 200 PG size exclusion column (GE Healthcare, United States) with buffer containing 25 mM Tris–HCl pH 8.0, 150 mM NaCl to 95% purity.

### Crystallization of the Apo-Mesaconyl-Coenzyme A Transferase and Heavy Atom Soaking of the Crystals

The purified MCT protein was concentrated to 20 mg mL^–1^ at 4°C using an Amicon Ultra centrifugal filter device (10 kDa molecular-weight cutoff; Millipore, Germany). Protein concentration was determined using a NanoDrop (Thermo Scientific, United States) to measure the absorption at 280 nm. Crystallization was performed using the hanging-drop vapor diffusion method, with 1.5 μL of protein sample mixed with an equal volume of the reservoir solution, and then the drop was equilibrated against 200 μL reservoir solution. Hexagonal shaped crystals were obtained at 14 mg mL^–1^ with the reservoir solution (14% PEG4000, 0.1M Li_2_SO_4_, 0.1M Sodium Citrate) at 16°C. In order to determine the phase using single-wavelength anomalous dispersion data, heavy atom soaking of the crystals was performed. The purified MCT sample was mixed with various heavy atom derivatives from the heavy atom screen kit (Hampton Research, United States) at different concentrations. The homogeneity and binding properties of the mixed samples were analyzed by Native-PAGE. Then the derivatives represent the higher homogeneity with the MCT protein were selected for soaking. Finally, the native-MCT crystals were transferred and soaked in a solution containing 14% PEG4000, 0.1M Li_2_SO_4_, 0.1 M Sodium Citrate, 2.5 mM phenylmercury acetate, or 2.5 mM ethylmercurithiosalicylic acid, sodium salt for soaking.

### Crystal Data Collection, Structure Determination, and Refinement

The optimized native-MCT crystals and Hg-soaked derivative crystals were cryo-protected by adding 25% glycerol to the reservoir solution and flash-frozen with liquid nitrogen, respectively. A 2.5 Å resolution dataset of native-MCT was collected at SSRF beamline BL19U1 with a 400 mm crystal-to-detector distance at wavelength of 0.97892 Å. 360 diffraction frames were collected with 1° oscillation per image. The crystal belongs to space group *C2* with unit cell dimensions *a* = 364.413 Å, *b* = 210.118 Å, *c* = 73.476 Å, α = 90°, β = 95.812°, γ = 90°. The diffraction data of Hg-soaked derivative crystal was also collected at SSRF BL19U1 with a 400 mm crystal-to-detector distance at wavelength of 0.87250 Å and 720 diffraction frames were collected with 0.5° oscillation per image. A 2.9 Å dataset was collected belonging to space group *C2* with unit cell dimensions *a* = 363.824 Å, *b* = 209.727 Å, *c* = 73.479 Å, α = 90°, β = 95.535°, γ = 90° (Table 1).

**TABLE 1:** Data collection and processing statistics of *R. castenholzii* MCT.

Crystal sample	Native MCT	Hg-derivative of MCT
Diffraction source	BL19U1, SSRF	BL19U1, SSRF
Wavelength (Å)	0.97892	0.87250
Temperature (K)	100	100
Detector	Pilatus3 6M	Pilatus3 6M
Crystal-to-detector distance (mm)	400	400
Rotation range per image (°)	1	0.5
Total rotation range (°)	360	360
Exposure time per image (s)	0.2	0.3
Space group	*C2*	*C2*
Cell parameters (Å)	*a* = 364.413,	*a* = 363.824,
	*b* = 210.118,	*b* = 209.727,
	*c* = 73.476	*c* = 73.479
	α = 90°, β = 95.812°,	α = 90°, β = 95.535°,
	γ = 90°	γ = 90°
Resolution range (Å)	50–2.50 (2.54-2.50)	50–2.90 (2.95-2.90)
Total no. of reflections	1300633	994583
No. of unique reflections	188922 (9443)	120126 (5862)
Completeness (%)	99.7 (99.2)	98.8 (95.9)
Redundancy	6.9 (6.6)	8.3 (7.8)
<*I*/σ(*I*)>	15.82 (3.95)	9.83 (2.17)
CC_1/2_	0.956 (0.902)	0.950 (0.864)
*R*_merge_ (%)	14.7 (45.4)	17.6 (67.7)
Overall *B* factors from Wilson plot (Å^2^)	24.43	26.63
**Matthews coefficient**		
V_M_ (Å^3^ Da^–1^)	5.08	4.95
Solvent content (%)	75.81	75.15

*R_merge_ = Σ_hkl_ Σ_i_—I_i_(hkl)-<I(hkl)>—/Σ_hkl_ Σ_i_ I_i_(hkl), where I_i_(hkl) is the intensity of the ith measurement of reflection hkl and <I(hkl)> is the mean intensity of all symmetry related reflection.*

Diffraction data were processed, integrated, and scaled with HKL3000R software (Minor et al., 2006). The data quality was assessed using SFCHECK (Vaguine et al., 1999), and the solvent content was calculated using MATTHEWS_COEF from CCP4 (Matthews, 1968; Collaborative Computational Project, 1994). The dataset of Hg-soaked derivative crystal was used for phasing by single-wavelength anomalous dispersion (SAD) method, the positions of Hg-atoms were determined and the initial model was automatically built by AutoSol program (Terwilliger et al., 2009) and initially refined by Phenix-Refine (Afonine et al., 2012). Then the structure model was built using Coot (Emsley and Cowtan, 2004) and refined against the native dataset to 2.5 Å resolution using Refmac5 (Murshudov et al., 1997) from CCP4 package (Collaborative Computational Project, 1994) and the Phenix (Liebschner et al., 2019; Table 1).

### Gel Filtration and Sedimentation Velocity Analytical Ultracentrifugation

Gel filtration and sedimentation velocity analytical ultracentrifugation (AUC) were performed to check the oligomerization state of MCT in solution. The purified MCT sample was loaded on a Superdex 200 10/300 GL size exclusion column (GE Healthcare, United States) and eluted with buffer containing 25 mM Tris–Cl pH 8.0, 150 mM NaCl at 0.5 mL min^–1^. Sedimentation experiments were performed on a Beckman Coulter ProteomeLab XL-I ultracentrifuge (Beckman Coulter, Indianapolis, United States) using a 4-hole An-60Ti rotor. Samples with an initial absorbance at 280 nm of approximately 0.5–0.8 were equilibrated for 2 h at 20°C under a vacuum prior to sedimentation. The absorbance at 280 nm was measured using a continuous scan mode during sedimentation at 55,000 rpm in 12 mm double-sector cells. The data were analyzed using sedfit (Schuck, 2000).

### Molecular Dynamics Simulation and Binding Free Energy Calculations

The binding structure of MCT and CoA was constructed through structure superimposition with the formyl-CoA transferase from *Oxalobacter formigenes* (FRC, PDB ID: 1P5R) (Ricagno et al., 2003), a member of the type III CoA transferase. Based on the complex structure of MCT-CoA, the binding structures of mesaconyl-C1-CoA and mesaconyl-C4-CoA were generated under criteria that the mesaconyl-C1 and mesaconyl-C4 groups form as much as possible electrostatic and hydrogen bonding interactions with the surrounding amino acid residues of MCT. Structure superimposition and editing were accomplished by using the UCSF Chimera software (Pettersen et al., 2004).

Molecular dynamics (MD) simulations were performed by using the AMBER 12 software (Case et al., 2005). The CoA, mesaconyl-C1-CoA and mesaconyl-C4-CoA bound MCT structures were individually immersed into the center of a truncated octahedron box of TIP3P water molecules with a margin distance of 10.0 Å. The environmental sodium counterions were added to keep the system in electric neutrality. The AMBER ff14SB force field was applied for MCT (Maier et al., 2015). For the CoA, mesaconyl-C1-CoA and mesaconyl-C4-CoA, structure optimization was firstly performed with Gaussian 03 at the level of DFT B3LYP/6-31G(d) (Wang et al., 2022), then their atomic partial charges were calculated using the restricted electrostatic potential (RESP) method with a basis set of HF/6-31G(d) (Bayly et al., 1993). The other force field parameters of CoA analogs were generated from the Generalized Amber Force field (GAFF) with the Antechamber module of Amber Tools (Wang et al., 2004). Finally, by following the same procedure in our previous report (Wang et al., 2019), MD simulations were conducted with varied time scales, i.e., 200 ns for MCT-CoA and 300 ns for MCT-mesaconyl-C1-CoA/mesaconyl-C4-CoA binding structures.

Upon the equilibrium of MD simulations, the binding free energies (ΔG_bind_) between MCT and the bound CoA analogs were obtained through the molecular mechanics/generalized Born surface area (MM/GBSA) calculation approach (Kollman et al., 2000):


(1) $$\Delta G_{\mathrm{bind}}=G_{\mathrm{complex}}-\left(G_{\mathrm{protein}}+G_{\mathrm{ligand}}\right)$$

(2) $$\Delta G_{\mathrm{bind}}=\Delta H-T\Delta S\approx \Delta E_{\mathrm{MM}}+\Delta G_{\mathrm{solv}}-T\Delta S$$

(3) $$\Delta E_{\mathrm{MM}}=\Delta E_{\mathrm{vdW}}+\Delta E_{\mathrm{ele}}+\Delta E_{\mathrm{ini}}$$

(4) $$\Delta G_{\mathrm{solv}}=\Delta G_{\mathrm{GB}}+\Delta G_{\mathrm{SA}}$$

where E_MM_ is the gas phase interaction energy comprising van der Waals energy (E_vdW_) and electrostatic energy (E_ele_). Internal strain energy difference (ΔE_int_) equals zero since no contribution difference of bond, angle and torsion takes place. G_solv_ is the solvation free energy, including the contributions form a polar part (G_GB_) and a non-polar part (G_SA_). ΔG_GB_ was estimated using the generalized Born model with the interior and exterior dielectric constants set to 4 and 80, respectively (Liu et al., 2018). ΔG_SA_ was estimated using the LCPO algorithm: ΔG_SA_ = γΔSASA + β, where γ and β were set to 0.0072 and 0, respectively (Weiser et al., 1999). 200 snapshots were evenly extracted from the last 40 ns of each MD trajectory for the calculations of ΔE_vdW_, ΔE_ele_, ΔG_GB_ and ΔG_SA_. The solute entropy term TΔS is sometimes approximated by normal mode entropy (Genheden and Ryde, 2015), but such treatment rarely leads to improvement in the correlation with experiments (Deng et al., 2019). In this work we do not include the solute entropy term in estimating ΔG_bind_.

## Results

### Overall Structure of Mesaconyl-Coenzyme A Transferase From *Roseiflexus castenholzii*

**FIGURE 1:**
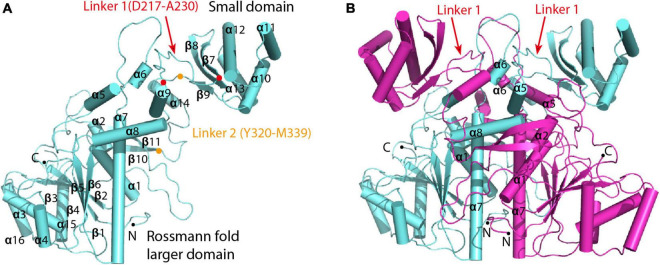
Overall crystal structure of *R. castenholzii* MCT. **(A)** Overall structure of the subunit that comprises a dimer of MCT. Each subunit consists of a Rossmann fold larger domain and a small domain, which are joined together by two linker regions (Residues D217-A230 for linker 1 and Y320-M339 for linker 2). The N- and C-terminus, and amino acid residues located at the two ends of the linker are indicated as dots. **(B)** The conformation of the dimer. Two subunits are cross interlocked at the linker regions, wherein α5, α6, α7, and α8 from each subunit symmetrically interacted in the center of the dimer interface.

To investigate the catalytic mechanism of MCT, we determined the crystal structure of apo-MCT using the single wavelength anomalous dispersion (Hendrickson et al., 1985; Rice et al., 2000) data from the crystals soaked with Hg-derivatives. The final model was refined to an *R*_*work*_ of 15.9% and an *R*_*free*_ of 19.2% at 2.5 Å resolution (Tables 1, 2). The structure covers the full-length enzyme (Met1–Glu407) that consists of a Rossmann fold larger domain (Residues M1-N216 and F340-E407) and a small domain (Residues F231-P319), which are joined together by two linker regions (Residues D217-A230 for linker 1 and Y320-M339 for linker 2) (Figure 1A and Supplementary Figure 1). Two MCT subunits are cross interlocked at the linker regions, wherein α5 (D135-L142), α7 (A163-T186), and α8 (L196-L207) from each subunit are symmetrically interacted in the center, with two small domains and two Rossmann fold larger domains distributed at the top and bottom sides, respectively (Figure 1B). The dimer interface is stabilized by extensive hydrogen bonding and salt bridge interactions with amino acid pairs Arg324–Asp219 (3.0 Å), Arg324–Asp217 (3.3 Å), Tyr320-Asn158 (2.6 Å), Asp330-Lys197 (2.8 Å), and so on, as well as hydrophobic interactions involve side chains of Tyr136-Tyr320-Leu227, Ile166-Val167, and Met171 and so on (Supplementary Figure 2A). Both gel filtration and ultracentrifugation analyses showed that *R. castenholzii* MCT exists as a dimer in solution (Supplementary Figures 2B,C), which is consistent with previous biochemical evidences that the type III CoA transferases function as a dimer.

**TABLE 2:** Structural refinement statistics of *R. castenholzii* MCT.

Resolution (Å)	50–2.5
No. of unique reflections	188472 (18766)
Reflections used for *R*_free_	9360 (876)
*R*_work_ (%)	15.9
*R*_free_ (%)	19.2
No. of atoms	20538
macromolecules	18792
ligands	0
solvent	1746
Protein residues	2435
R.M.S. deviations	
bonds (Å)	0.008
angles (°)	1.02
Ramachandran favored (%)	98.39
Ramachandran allowed (%)	1.61
Ramachandran outliers (%)	0.00
Rotamer outliers (%)	1.04
Clashscore	3.22
Average B-factor (Å^2^)	30.53
macromolecules	30.17
solvent	34.39

The *C2* crystal of apo-MCT contains a hexamer of three dimers in one asymmetric unit. The small domains of three dimers are closely associated with each other at α11 (T263-L273) and α12 (E280-A300) helices that are related by a threefold rotational axis (Supplementary Figure 3A). The loop regions (T148-A163) between α6 and α7 of six subunits are directing into the center, forming a cavity with a diameter about 14 Å at the threefold axis. At the interface, the guanidine side chains of Arg285 and the main chain oxygen of Ala300 form weak hydrogen bonding interactions, and π-π stacking interactions are formed between the guanidine and imidazole side chains of Arg288 and His299 (Supplementary Figure 3B). The PISA analyses (Krissinel and Henrick, 2007) revealed a CSS core of 1.0 and an average interface area of 6675.8 Å^2^ for the basic dimer unit as shown in Figure 1B, which are contributed by the interactions from 74 hydrogen bonds and 43 salt bridges. Theoretical calculations predict a free energy of interaction (ΔG^int^) of -94.0 kcal mol^–1^ and dissociation (ΔG^diss^) of 115.9 kcal mol^–1^ for the dimer. Whereas the average interface area of other dimer forms ranged from 88.5 to 392.1 Å^2^, with a CSS score from 0.011 to 0.044 (Supplementary Table 1). Therefore, the basic unit of dimer assembly shown in Figure 1B represents the stable biological assembly, and the hexamer assembly is formed due to the crystal packing.

**FIGURE 2:**
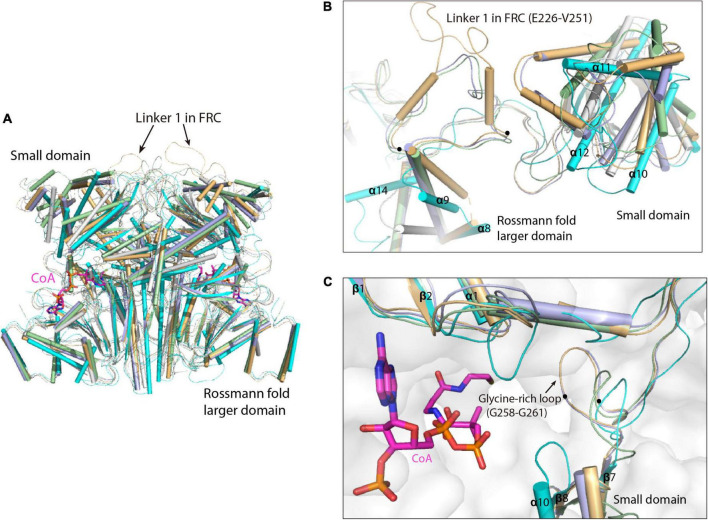
Superimposition of *R. castenholzii* MCT dimer with that of the type III CoA-transferases. **(A)** Overall structure superimposition of MCT dimer with the dimers of formyl-CoA transferase from *Oxalobacter formigenes* (FRC, PDB ID: 2VJQ, yellow orange); formyl-CoA transferase from *E. coli* (YfdW, PDB ID: 1PT5, light blue); CoA transferase III from *Mycobacterium tuberculosis* (Rv3272, PDB ID: 5YIT, white); formyl-CoA: oxalate-CoA transferase from *Acetobacter aceti* (FCOCT, PDB ID: 3UBM, light green). The structures are aligned at the Rossman fold larger domain. The linker 1 in FRC is indicated with arrows. The CoA molecules bound in FCOCT are shown as stick models in magenta. **(B)** Comparison of the small domains and linker 1 loops in MCT and some type III CoA-transferases. The linker 1 loop in FRC is indicated with dots at the two ends of the loop and labeled. **(C)** Comparison of the substrate binding pocket of MCT with that of the type III CoA-transferases. The glycine-rich loop in FRC is indicated with dots at the two ends of the loop and labeled.

Superimposition of the MCT dimer with that of the type III CoA-transferases reveals high structural similarity at the Rossmann fold larger domains, whereas the conformations of the small domain are dramatically different (Figure 2A). This is consistent with the multiple sequence alignment that showed considerable amino acid sequence conservation in the Rossman fold larger domains but less conservation in the small domains (Supplementary Figure 1). Compared to the formyl-CoA transferase from *O. formigenes* (FRC, PDB ID: 2VJQ) (Ricagno et al., 2003) that contain much longer linker 1 loop (Residues E226-V251), the corresponding loop are shorter in the formyl-CoA: oxalate-CoA transferase from *Acetobacter aceti* (FCOCT, PDB ID: 3UBM) (Mullins et al., 2012) and the formyl-CoA transferase from *E. coli* (YfdW, PDB ID: 1PT5) (Gogos et al., 2004; Toyota et al., 2008; Figure 2B; Supplementary Figure 1). In contrast, absence of this loop in MCT leads to the removal of a glycine-rich loop (G258-G261) that points directly into the active site pocket of FRC and Yfdw, as well as the shorter glycine-rich loop in FCOCT, creating a relatively larger substrate binding pocket of MCT (Figure 2C).

### Alternative Conformations of *Roseiflexus castenholzii* Mesaconyl-Coenzyme A Transferase in Binding With Different Coenzyme A Analogs

**FIGURE 3:**
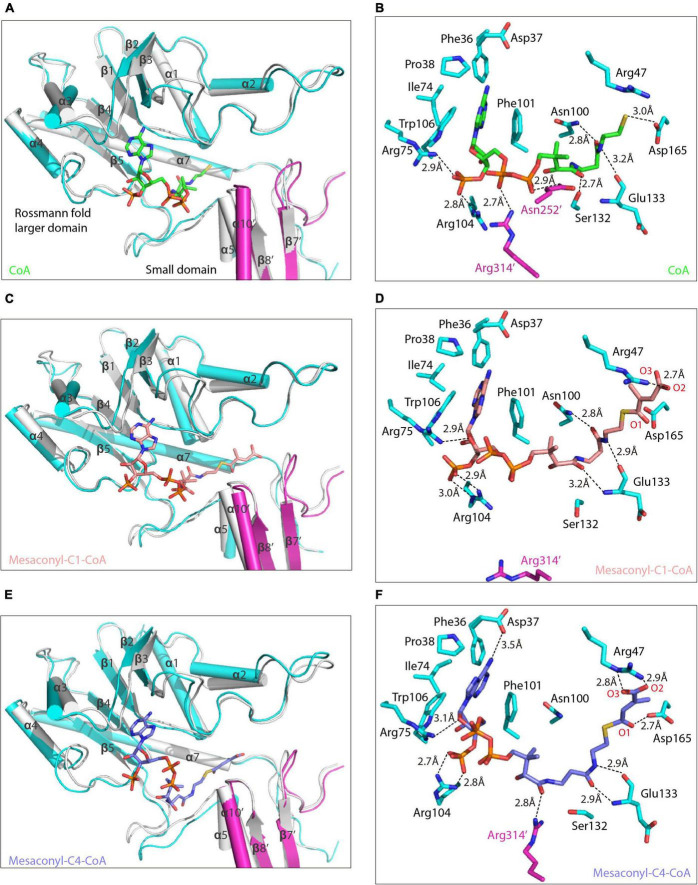
Alternative conformations of the substrate binding pocket in binding with different CoA analogs. **(A,C,E)** The equilibrated binding structures of MCT in complex with CoA (**A**, green), mesaconyl-C1-CoA (**C**, salmon), and mesaconyl-C4-CoA (**E**, blue) are superimposed with the crystal structure of apo-MCT (the two subunits of the binding structures are colored in cyan and magenta). The secondary structures that form the substrate binding pocket of MCT are shown in ribbon and labeled, and the CoA analogs are shown in stick models. **(B,D,F)** Coordination of three CoA analogs with the substrate binding pocket of MCT. Amino acid residues essential for coordinating the CoA (**B**, green), mesaconyl-C1-CoA (**D**, salmon), and mesaconyl-C4-CoA (**F**, blue) are shown as stick models, the hydrogen bonding interactions are shown as dashed lines with distances labeled.

Attempts to obtain the substrate-bound structure of *R. castenholzii* MCT were not successful, by either co-crystallization or soaking the crystals with various CoA analogs (succinyl-CoA, acetyl-CoA, propionyl-CoA, malonyl-CoA), or the reaction intermediate mesaconic acid. To explore the catalytic mechanism of MCT, the binding structures of MCT with substrate analog CoA, the reaction substrate mesaconyl-C1-CoA and product mesaconyl-C4-CoA were generated, and their binding features were characterized through molecular dynamics (MD) simulations. No drastic fluctuations occurred at the root-mean-square deviation (RMSD) values during MD simulations, indicating the conformations of these complex structures are rather stable (Supplementary Figures 4A–C). In addition, the root-mean square fluctuation (RMSF) profiles of the binding complexes showed overall consistent pattern to that of the crystal structure of apo-MCT, indicating binding of the substrate analogs did not cause drastic conformational changes to MCT (Supplementary Figure 4D). Two CoA molecules were initially docked into the dimer of MCT through structural superimposition with the (FRC, PDB ID: 1P5R) (Ricagno et al., 2003), which gives a main chain RMSD of 2.378 and results in binding of the CoA molecules at the interface of the Rossmann fold larger domain from one subunit and the small domain from the other subunit (Figure 3A).

In the equilibrated binding structures derived from MD simulations, the adenine ring of the CoA is buried in a pocket formed by several loop regions connecting α3-β3, α2-β2, α1-β1 from top and α4-β4 at the bottom, wherein it is surrounded by the side chains of Pro38, Phe36, Ile72, Trp106, and Phe101 (Figures 3A,B). The phosphate moiety is hydrogen bonded with Arg75 NH2 (2.9 Å) and Arg104 NH2 (2.8 Å), respectively. The CoA pantetheine O1 and O4 atoms are hydrogen bonded with the NH2 atoms of Arg314’ (2.7 Å) and Asn252’ (2.9 Å) from the small domain of the other subunit. On the other side, the pantetheine tail is directing into the cavity formed by β1-α1, β5-α5, and α6-α7, wherein it is stabilized by hydrogen bonding interactions with the NH2 of Asn100 (2.8 Å), the hydroxyl group of Ser132 (2.7 Å), and the main chain oxygen of Glu133 (3.2 Å). At the end of CoA, the thiol group forms one hydrogen bond with Asp165 OD2 (3.0 Å).

In the equilibrated mesaconyl-C1-CoA bound structure, the adenine ring adopts similar spatial environment as CoA. The ribose oxygen forms hydrogen bond with Arg75 NH2 (2.9 Å), the phosphate O7 (3.0 Å), and O8 (2.9 Å) form hydrogen bonds with the guanidine side chain of Arg104 (Figures 3C,D). The pantetheine tail of mesaconyl-C1-CoA is hydrogen bonding with NH2 of Asn100 (2.8 Å), and main chain nitrogen (3.2 Å) and oxygen (2.9 Å) of Glu133. Importantly, the mesaconic end is positioned into a slit formed by loop regions connecting α6-α7 and β7’-α9’, wherein it is coordinated by hydrogen bonding interactions with Arg47 NH1 (2.7 Å) and electrostatic interaction between the carbonyl C and Asp165 OD2 (3.4 Å) (Figures 3C,D and Supplementary Figure 5A).

**FIGURE 4:**
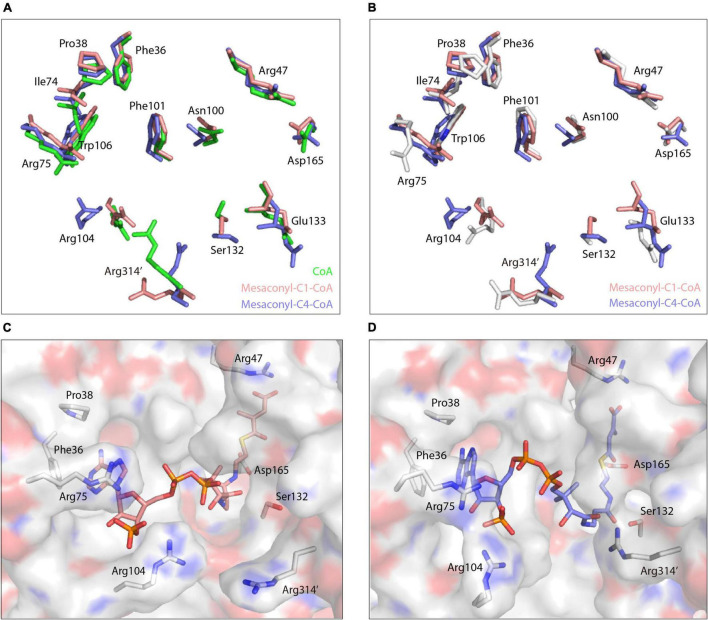
Conformational changes of the substrate binding pocket in binding with different CoA analogs. **(A)** Superimpositions of the amino acid residues that are necessary for coordinating the CoA (green), mesaconyl-C1-CoA (salmon), and mesaconyl-C4-CoA (blue). **(B)** Superimpositions of the amino acid residues that are necessary for coordinating mesaconyl-C1-CoA (salmon) and mesaconyl-C4-CoA (blue) with the apo-MCT (white). **(C,D)** The binding mode of mesaconyl-C1-CoA **(C)**, mesaconyl-C4-CoA **(D)**, and the surface electrostatic potentials of MCT that are represented with positive, negative, and neutral charged residues colored in blue, red, and white, respectively. The key amino acid residues for coordinating the CoA analogs are shown in stick models.

In the equilibrated binding structure with mesaconyl-C4-CoA (Figure 3E), the guanidine side chains of Arg104, Arg314’, and Ser132 undergo conformational changes as compared to the mesaconyl-C1-CoA bound MCT (Figures 4A,B). The adenine ring of mesaconyl-C4-CoA is coordinated in same conformation as mesaconyl-C1-CoA, except an additional hydrogen bond between the adenosine N6 atom and Asp37 OD2 (3.5 Å) (Figure 3F). Hydrogen bonding interactions are observed between the ribose oxygen and Arg75 NH2 (3.1 Å), the ribose phosphate oxygens with Arg104 NH1 of (2.8 Å) and NH2 (2.7 Å). Notably, the guanidine group of Arg314’ dramatically flipped toward the pantetheine tail of mesaconyl-C4-CoA and hydrogen bonded with O9 (2.8 Å). Additionally, the pantetheine tail are hydrogen bonded with the main chain oxygen (2.9 Å) and nitrogen (2.9 Å) of Glu133. Although the conformation of mesaconyl-C4-CoA changes in comparison with mesaconyl-C1-CoA, the mesaconic end maintains a similar binding mode with the surrounding Asp165 (2.7 Å) and Arg47 (2.9 Å) residues (Figure 3F).

### Binding Features Characterized From Molecular Dynamics Simulation

To further explore the binding characteristics of MCT with the CoA analogs, the binding affinities were evaluated based on the widely recognized molecular mechanics/generalized Born surface area (MM/GBSA) calculation (Kollman et al., 2000). As shown in Table 3, mesaconyl-C1-CoA exhibited the highest binding free energies to MCT (ΔG = –90.33 kcal mol^−1^), whereas CoA and mesaconyl-C4-CoA showed comparable binding free energies, indicating mesaconyl-C1-CoA is more favorable for binding to MCT. It is noted that electrostatic interaction (ΔE_ele_) and polar solvation effect (ΔG_GB_) contributed negatively and positively to the binding with CoA, respectively (Table 3). In contrast, the binding free energies of mesaconyl-C1-CoA and mesaconyl-C4-CoA were both contributed by a positive ΔE_ele_ and a negative ΔG_GB_. Considering the adenine ring and pantetheine tail of these CoA analogs are basically immobilized with similar hydrophobic and hydrogen bonding interactions (Figures 3B,D,F), these reverse effects are probably resulted from the ligated mesaconic groups.

**TABLE 3:** Binding free energies calculated using the MM/PBSA approach.

Binding complex	Energy component^a^				
	ΔE_ele_	ΔE_vdW_	ΔG_GB_	ΔG_SA_	ΔG
MCT-CoA	−20.45 ± 5.63	−68.66 ± 4.93	12.61 ± 3.41	−10.25 ± 0.24	−86.76 ± 4.29
MCT-mesaconyl-C1-CoA	40.37 ± 8.593	−77.72 ± 4.88	−40.92 ± 8.64	−12.06 ± 0.32	−90.33 ± 5.07
MCT-mesaconyl-C4-CoA	23.05 ± 7.69	−68.82 ± 4.83	−27.76 ± 7.09	−11.49 ± 0.24	−85.02 ± 3.90

*^a^Energies are in kcal mol^–1^.*

**FIGURE 5:**
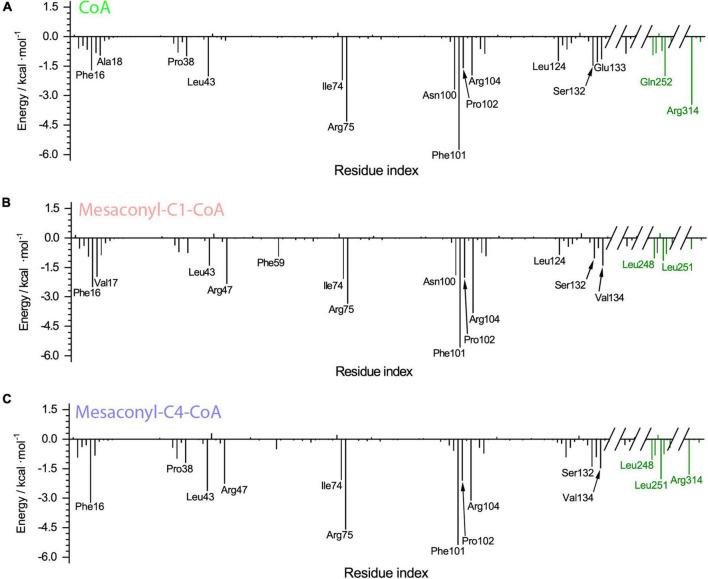
The per-residue decomposition of the binding free energies of *R. castenholzii* MCT in binding with CoA **(A)**, mesaconyl-C1-CoA **(B)**, and mesaconyl-C4-CoA** (C)**. The binding free energy values (kcal mol^–1^) are plotted against the amino acid numbers to show their contributions.

The binding free energy was further decomposed to identify the per-residue contribution (Figure 5). Phe16 contributed to the binding through hydrophobic interactions with the alkyl groups linked to the S atom of CoA analogs, more extensive hydrophobic contacts resulted in increased contribution of free energies to the binding of mesaconyl-C1-CoA and mesaconyl-C4-CoA with MCT (Figures 5A–C). Through electrostatic interactions with the phosphate group of CoA analogs, Arg75 and Arg104 made significant contributions to free energies that resulted in stable binding of the three analogs. The largest per-residue contribution came from Phe101, which forms parallel π-π interactions with the adenine ring of all the three CoA analogs. Notably, Arg47 made considerable contributions to the binding of mesaconyl-C1-CoA and mesaconyl-C4-CoA, but almost no contribution to the binding of CoA (Figures 5A–C). This agreed well with the observations that no electrostatic and hydrogen bonding interactions exist between Arg47 and the terminal thiol group of CoA, due to lack of the ligated mesaconic groups (Figures 3B,D,F). In contrast to the bindings of CoA and mesaconyl-C4-CoA, Arg314’ from the other subunit of the dimer showed negligible contribution to the binding of mesaconyl-C1-CoA, which is also consistent with the characterized binding conformations (Figures 3B,D,F, 5). Therefore, Arg47, Arg75, Phe101, and Arg104 are both essential for the binding of mesaconyl-C1-CoA and mesaconyl-C4-CoA, and the interaction with Arg314’ is pivotal for discriminating these two analogs.

Further superimposition analyses revealed that the side chains of Arg104 and Arg314’ undergo dramatic conformational changes in binding with the three CoA analogs (Figures 4A,B). Compared to the structure of apo-MCT, the guanidine side chain of Arg75 and Arg104 was flipped toward the substrate binding pocket to stabilize the ribose and phosphate group of the CoA analogs through hydrogen bonding interactions (Figures 3B,D,F, Figure 4B). For the binding of mesaconyl-C4-CoA, the guanidine group of Arg314’ was oscillated approximately 60° toward the pantetheine tail, the guanidine group of Arg104 was concomitantly flipped about 40° outward, these conformational changes together broaden the substrate entrance located in between the guanidine groups of Arg104 and Arg314’ (Figures 4B–D). In addition, binding of mesaconyl-C1-CoA and mesaconyl-C4-CoA also resulted in distinct conformations and surface electron potentials of the substrate binding pocket. Mesaconyl-C1-CoA was fitted into the substrate binding pocket with pantetheine tail and phosphate group exposed, whereas the pantetheine tail of mesaconyl-C4-CoA was completely buried within the pocket (Figures 4C,D).

### Molecular Mechanism of *Roseiflexus castenholzii* Mesaconyl-Coenzyme A Transferase Catalyzed Intramolecular Coenzyme A Transfer

The mesaconic end of mesaconyl-C1-CoA and mesaconyl-C4-CoA were stabilized by hydrogen bonding interactions with Asp165 and Arg47 (Figures 3D,F). In the equilibrated binding structure with mesaconyl-C1-CoA, no hydrogen bonding but electrostatic interaction was observed between the carbonyl C1 atom and the hydroxyl group (OD2) of Asp165 (3.4 Å), providing a prerequisite for hydrophilic attack at the C1 atom. On the other side, the O2 atom of the mesaconic end was immobilized in a chain of hydrogen bonding interactions with Arg47 NH1 (2.7 Å) and a water molecule (2.8 Å) (Supplementary Figure 5A). Similar hydrogen bonding interactions were also observed for the O2 atom of mesaconyl-C4-CoA. In contrast, mesaconyl-C4-CoA was accommodated with a longer distance between the C4 atom and Asp165 OD2 (3.7 Å) (Supplementary Figure 5B). Considering the strictly conservation of Asp165 with the catalytic aspartate residues of type III CoA-transferases, Asp165 and Arg47 residues are essential for catalyzing the CoA transfer reaction.

**FIGURE 6:**
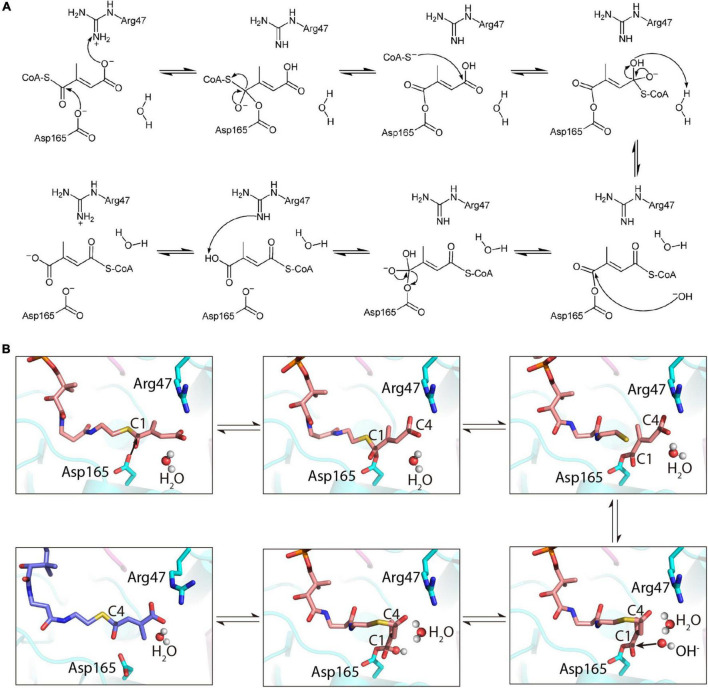
Illustration of the proposed mechanism of *R. castenholzii* MCT as an intramolecular CoA-transferase. **(A)** The proposed mechanism of MCT in catalyzing the reversible CoA transfer between mesaconyl-C1-CoA and mesaconyl-C4-CoA. **(B)** Cartoon presentation of the mechanism of MCT in catalyzing the intramolecular CoA transfer reaction. Mesaconyl-C1-CoA (salmon), mesaconyl-C4-CoA (blue), and the key amino acid residues Arg47 and Asp165 are shown in stick models, and the water molecules are shown as spheres.

Based on the crystal structure of apo-MCT and the characterized binding features with mesaconyl-C1/C4-CoA, we proposed a catalytic mechanism for the intramolecular CoA transfer reaction (Figures 6A,B). At the close-to-neutral pH environment of the cell, both Asp165 and mesaconyl-C1-CoA are deprotonated, and Arg47 is protonated. Initially, the negatively charged carboxyl oxygen of Asp165 launches a nucleophilic attack at the carbonyl C1 atom of mesaconyl-C1-CoA, and a proton involved in the electrostatic and hydrogen bonding interactions at the reactant state is concomitantly extracted from the guanidine group of Arg47 to the terminal carboxyl group of mesaconyl-C1-CoA. Subsequent intramolecular electron reorganization releases the CoAS^–^ and forms an enzyme-bound anhydride. The liberated CoAS^–^ anion is then engaged in a second nucleophilic attack at the carboxyl C4 atom of mesaconyl-C1-CoA. With involvement of a water molecule, the resultant tetrahedral intermediate is further converted to an enzyme (Asp165)-bound CoA-thioester, generating hydroxide ion and a new water. Subsequently, the released hydroxide ion initiates a third nucleophilic attack at the carbonyl C1 atom of the enzyme-bound CoA-thioester, then the electron rearrangement dissociates Asp165 from the CoA-thioester to its original deprotonated state. Finally, mesaconyl-C4-CoA is formed when a proton is extracted by the deprotonated guanine sidechain of Arg47 (Figures 6A,B).

## Discussion

Coenzyme A transferases are essential enzymes for the metabolism of carboxylic acids in all organisms, wherein they catalyze the reversible CoA transfer reactions with broad substrate specificity. In this study, we determined the crystal structure of a novel intramolecular CoA transferase, Mesaconyl-CoA C1-C4 CoA transferase (MCT) from a green non-sulfur photosynthetic bacteria *R. castenholzii*, and characterized its catalytic mechanisms through structural analyses and MD simulations. The basic dimer unit observed in the crystal structure is consistent with the findings that *R. castenholzii* MCT exists as a dimer in solution. The substrate mesaconyl-C1-CoA and product mesaconyl-C4-CoA are coordinated at the interface between the Rossman fold larger domain from one subunit and the small domain from the other subunit of the dimer. In the equilibrated structures, Arg47 made considerable contributions to the binding of mesaconyl-C1-CoA and mesaconyl-C4-CoA, whereas Arg314’ from the other subunit undergo dramatic conformational changes in binding with mesaconyl-C4-CoA. Together with Arg47 and one water molecule, a strictly conserved Asp165 residue are essential for catalyzing the reversible intramolecular CoA transfer reaction, through electrostatic and the hydrogen bonding interactions with the mesaconic tail of both the substrate and product.

Mesaconyl-coenzyme A transferase was originally identified in the 3-hydroxypropionic acid (3-HP) cycle of *C. aurantiacus* (Pierson and Castenholz, 1974), the representative bacteria of filamentous anoxygenic phototrophs (FAPs). FAPs are phylogenetically distant from other anoxygenic photosynthetic bacterium and form the deepest branch of photosynthetic bacteria (Oyaizu et al., 1987). This type of organism has acquired a “chimeric” photosynthetic system during evolution, with their reaction center and associated antenna resembling that of purple bacteria, whereas the light harvesting apparatus is similar as that of green sulfur bacteria (Blankenship, 2010; Hohmann-Marriott and Blankenship, 2011; Tang et al., 2011). Notably, FAPs evolved a new type carbon fixation pathway 3-hydroxypropionic acid bicycle, instead of Calvin cycle during evolution. This autotrophic pathway directly generates acetyl-CoA and pyruvate, and assimilates small organic fermentation products, such as acetate, propionate, or succinate even under anaerobic conditions. Notably, the byproduct 3-HP is an intermediate for the metabolism of dimethylsulfoniopropionate (Ansede et al., 1999; Sunda et al., 2002; Yoch, 2002), a ubiquitous osmoprotectant and antioxidant of algae (Todd et al., 2007; Johnston et al., 2008). Therefore, investigation of the enzymes in 3-HP cycle is necessary for understanding the autotrophic carbon fixation mechanisms of early branch prokaryotic phototrophs.

Multiple sequence alignment of *R. castenholzii* MCT with the type III CoA-transferases showed considerable amino acid sequence conservation in the Rossman fold larger domains but less conservation in the small domains, which is consistent with the structural superimposition analyses (Figure 2 and Supplementary Figure 1). These type III CoA-transferases are mostly involved in anaerobic biochemical pathways, wherein they play essential roles for activating specific organic acids for subsequent decarboxylation, β-oxidation or elimination of α- or β-hydroxy groups (Heider, 2001). The reaction mechanism of type III CoA transferases requires formation of covalently bound substrate intermediates with a conserved aspartate residue of the enzyme (Gruez et al., 2003; Ricagno et al., 2003; Gogos et al., 2004; Stenmark et al., 2004; Toyota et al., 2008). In the reaction of butyrobetainyl-CoA:(*R*)-carnitine-CoA transferase (CaiB), Asp169 initiates a nucleophilic attack at the substrate γ-butyrobetaine-CoA to form a covalently bound intermediate and release the CoAS^–^. Subsequently, the second substrate carnitine reacts with the covalent intermediate to form a reactant state, in which carnitine is also covalently bound to Asp169. This reactant state is stabilized by the release of γ-butyrobetaine. Then the CoAS^–^ anion launches a second nucleophilic attack at the enzyme-anhydride intermediate to produce carnityl-CoA and regenerate the deprotonated Asp 169 (Stenmark et al., 2004). In *R. castenholzii* MCT, the catalytic residue Asp165 is strictly conserved with CaiB (Stenmark et al., 2004), the formyl-CoA transferase from *O. formigenes* (FRC) and *E. coli* (YfdW) (Gogos et al., 2004; Toyota et al., 2008), as well as formyl-CoA: oxalate- CoA transferase from *A. aceti* (FCOCT) (Mullins et al., 2012) and a CoA transferase III from *Mycobacterium tuberculosis* (Rv3272) (Karade et al., 2019; Supplementary Figure 1), indicating MCT probably adopts a similar reaction mechanism as the type III CoA-transferase.

However, MCT catalyzes the reversible transformation of mesaconyl-C1-CoA to mesaconyl-C4-CoA, the only difference of the substrate and product of this reaction is the position of a methyl group. Compared to FRC that contain a much longer linker 1 loops, absence of this region in MCT leads to the removal of a glycine-rich loop that points directly into the active site pocket of FRC, YfdW, and FCOCT, results in a larger substrate binding pocket of MCT (Figures 2B,C), which could provide enough space for the intramolecular CoA transfer reactions. Based on the MD simulations, mesaconyl-C1-CoA exhibited higher binding free energies, indicating mesaconyl-C1-CoA is more favorable for binding to MCT. In the equilibrated structures, the mesaconic tails of both mesaconyl-C1-CoA and mesaconyl-C4-CoA were stabilized by a chain of hydrogen bonding interactions with Arg47 NH1 and one water molecule (Supplementary Figures 5A,B). Particularly, electrostatic interactions between the carbonyl C1 atom and Asp165 OD2 provided a prerequisite for hydrophilic attack of the reaction. Therefore, we proposed a previously unrecognized catalytic mechanism that involves formation of covalently bound intermediates with Asp165, and the participation of the less conserved Arg47 and water molecule. In this reaction mechanism, the water molecule takes place the role of the second substrate in type III CoA-transferases, providing protons for the enzyme-bound CoA-thioester and launching a nucleophilic attack to release the Asp165 and generate mesaconyl-C4-CoA. Therefore, the close proximities of the catalytic triad Asp165, Arg47, and a water molecule inside the active site pocket ensured occurrence of the reversible CoA-transfer reaction within only one substrate.

In conclusion, this study revealed the catalytic mechanism of an uncommon intramolecular CoA-transfer reaction in the 3-HP cycle of FAPs. As ancient anoxygenic photosynthetic bacteria, FAPs evolved an autotrophic bicycle to ensure sufficient utilization of the reaction intermediates for metabolism. Additionally, many multi-functional enzymes constitute an efficient carbon fixation system of the 3-HP cycle, in total 19 reactions are catalyzed by only 13 enzymes. Therefore, the intramolecular CoA transfer activity of MCT is also an economical and energy saving approach for efficient CoA shift of this autotrophic carbon fixation pathway. Besides the physiological importance, the mechanism of MCT in catalyzing intramolecular CoA transfer reaction can be applied to enzyme engineering or manipulations of the 3-HP cycle for synthesis of fine chemicals and important metabolites in the future.

## Data Availability Statement

The datasets presented in this study can be found in online repositories. The names of the repository/repositories and accession number(s) can be found in the article/Supplementary Material.

## Author Contributions

XX initiated the project and supervised all experiments. ZM and XZ performed expression, purification, crystallization, and data collection of *Roseiflexus castenholzii* MCT. CF and WW determined and refined the crystal structure. YX constructed the plasmid for recombinant expression of MCT. ZW performed the MD simulation and free energy calculations. ML and KW performed the gel filtration and AUC analyses. YH performed the PISA analyses. XX, ZW, and XWZ analyzed the data and wrote the manuscript. All authors contributed to the article and approved the submitted version.

## Conflict of Interest

The authors declare that the research was conducted in the absence of any commercial or financial relationships that could be construed as a potential conflict of interest.

## Publisher’s Note

All claims expressed in this article are solely those of the authors and do not necessarily represent those of their affiliated organizations, or those of the publisher, the editors and the reviewers. Any product that may be evaluated in this article, or claim that may be made by its manufacturer, is not guaranteed or endorsed by the publisher.

## Abbreviations

CoA: Coenzyme A
MCT: mesaconyl-CoA C1-C4 CoA transferase
FAP: filamentous anoxygenic phototroph
*C. aurantiacus*: *Chloroflexus aurantiacus*
*R. castenholzii*: *Roseiflexus castenholzii*
3-HP: 3-hydroxypropionate
MD: molecular dynamics
RMSD: root-mean-square deviation
RMSF: root-mean-square fluctuation
SAD: single-wavelength anomalous dispersion.
