# Supplementary material for: Crystal Structure of an Intramolecular Mesaconyl-Coenzyme A Transferase From the 3-Hydroxypropionic Acid Cycle of Roseiflexus castenholzii
Source: Front Microbiol. 2022 May 26;13:923367. doi: 10.3389/fmicb.2022.923367 (PMC9196870; doi:10.3389/fmicb.2022.923367)
Supplement: Supplementary file 1 [file Data_Sheet_1.pdf]

## *Supplementary Material for*

# Crystal Structure of an Intramolecular Mesaconyl-Coenzyme A Transferase From the 3-Hydroxypropionic Acid Cycle of *Roseiflexus castenholzii*


**Zhenzhen Min[1†], Xin Zhang[1†], Wenping Wu[1], Yueyong Xin[2], Menghua Liu[1], Kangle Wang[1], Xingwei Zhang[1], Yun He[3], Chengpeng Fan[3*], Zhiguo Wang[1*], Xiaoling Xu[1, 2, 4*]**

[1]Department of Biochemistry and Molecular Biology, School of Basic Medical Sciences, The Affiliated Hospital of Hangzhou Normal University, Hangzhou, China

[2]Photosynthesis Research Center, College of Life and Environmental Sciences, Hangzhou Normal University, Hangzhou, China

[3]Department of Biochemistry and Molecular Biology, School of Basic Medical Sciences, Wuhan University, Wuhan, China

[4]Key Laboratory of Aging and Cancer Biology of Zhejiang Province, Hangzhou Normal University, Hangzhou, China

[†]These authors contributed equally to this work and share first authorship.

*** Correspondence:**
Xiaoling Xu (xuxl@hznu.edu.cn), Zhiguo Wang (zhgwang@hznu.edu.cn) and Chengpeng Fan (chengpeng.fan@whu.edu.cn)

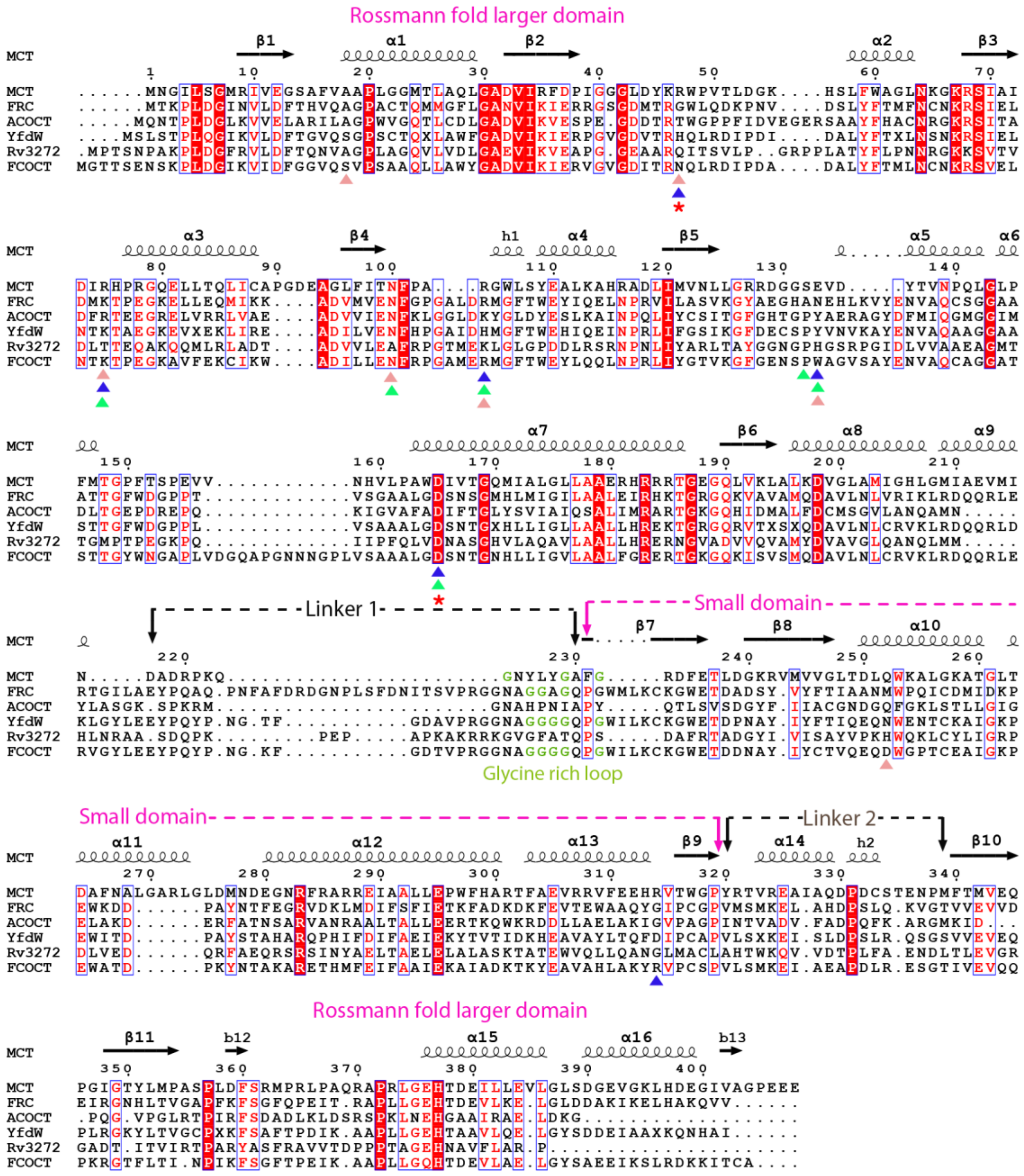

**Supplementary Figure 1.** Structure-based sequence alignments of *R. castenholzii* MCT with alternative type III CoA transferases. FRC, formyl-CoA transferase from *Oxalobacter formigenes* (PDB ID: 2VJQ); ACOCT, CAIB BAIF family protein from *Brucella suis* (PDB ID: 4ED9); YfdW, the formyl-CoA transferase from *E. coli* (PDB ID: 1PT5); Rv3272, CoA transferase III from *Mycobacterium tuberculosis* (PDB ID: 5YIT); FCOCT, formyl-CoA: oxalate-CoA transferase from *Acetobacter aceti* (PDB ID: 3UBM). The amino acids distributions of the Rossmann fold larger domain and the small domain are indicated on top of the sequences. The amino acid residues that are essential for the binding of CoA analogs are indicated by green triangles for CoA, salmon triangles for

mesaconyl-C1-CoA, and blue triangles for mesaconyl-C4-CoA. The key catalytic residues Arg47 and Asp165 are indicated by red asterisks, and the residues in glycine rich linker are shown in lime green.

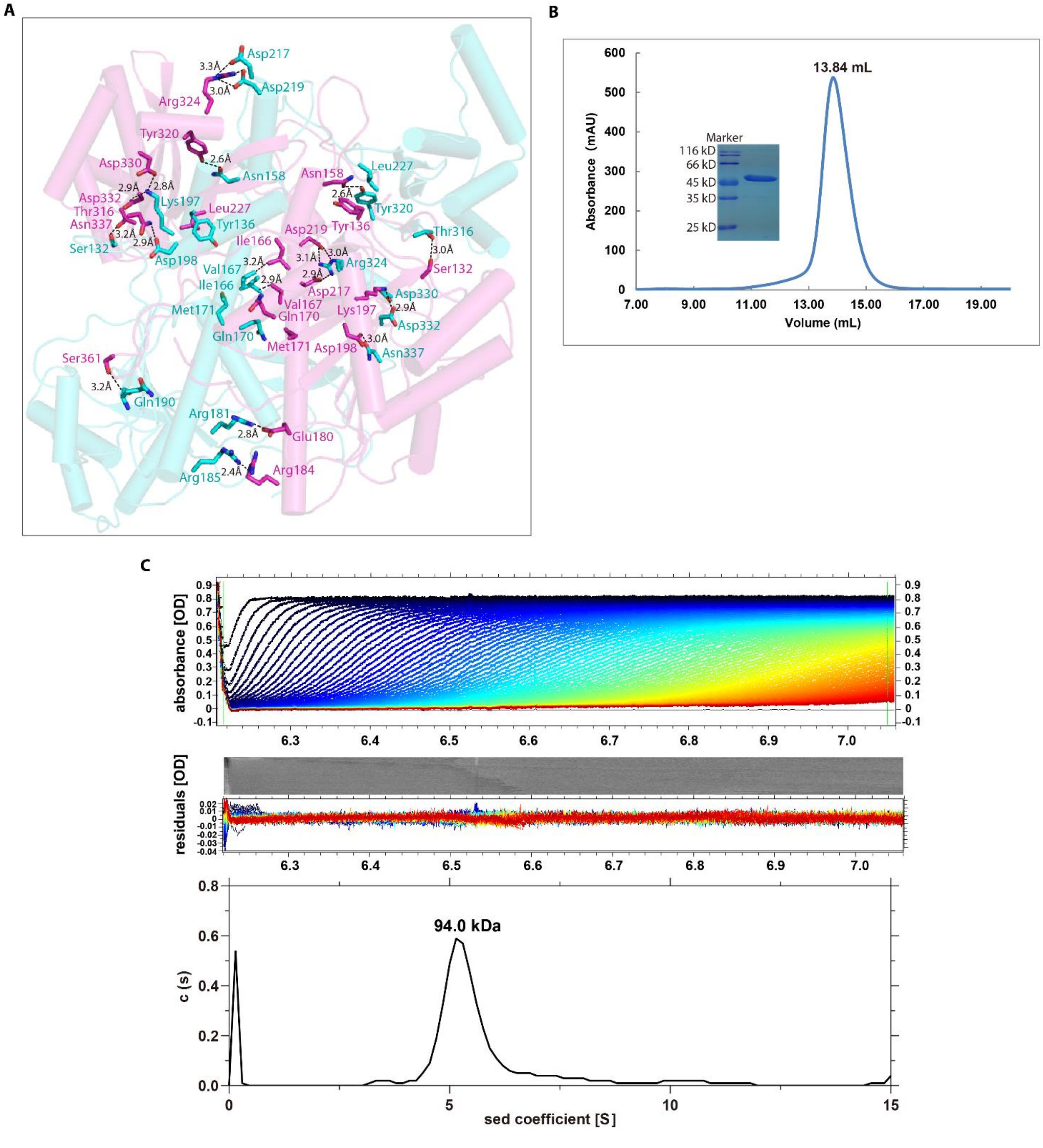


**Supplementary Figure 2.** Gel filtration and analytical ultracentrifugation (AUC) analyses indicated *R. castenholzii* MCT exists as a dimer in solution. **(A)** The hydrogen bonding and hydrophobic interactions at the dimer interface. Secondary structures from each subunit are shown in ribbon, the key amino acids essential for forming the dimer are shown in stick models, the hydrogen bonding interactions are shown as dashed lines with distances labeled. **(B)** Gel filtration and SDS-PAGE analyses of MCT. The absorption of MCT at 280 nm were recorded and the elutes were analyzed by SDS-PAGE. **(C)** Sedimentation velocity analytical ultracentrifugation (AUC) of MCT. The upper

panel shows the raw data from the time-course measurement of absorbance of the sample at 280 nm along the sample cell length. The middle panel shows residuals after fitting the data to the continuous size-distribution model. The lower panel shows the continuous mass distribution for the calculated solution. The results indicate that MCT exists as a dimer in solution (the calculated MW of MCT is 47, 152 Da).

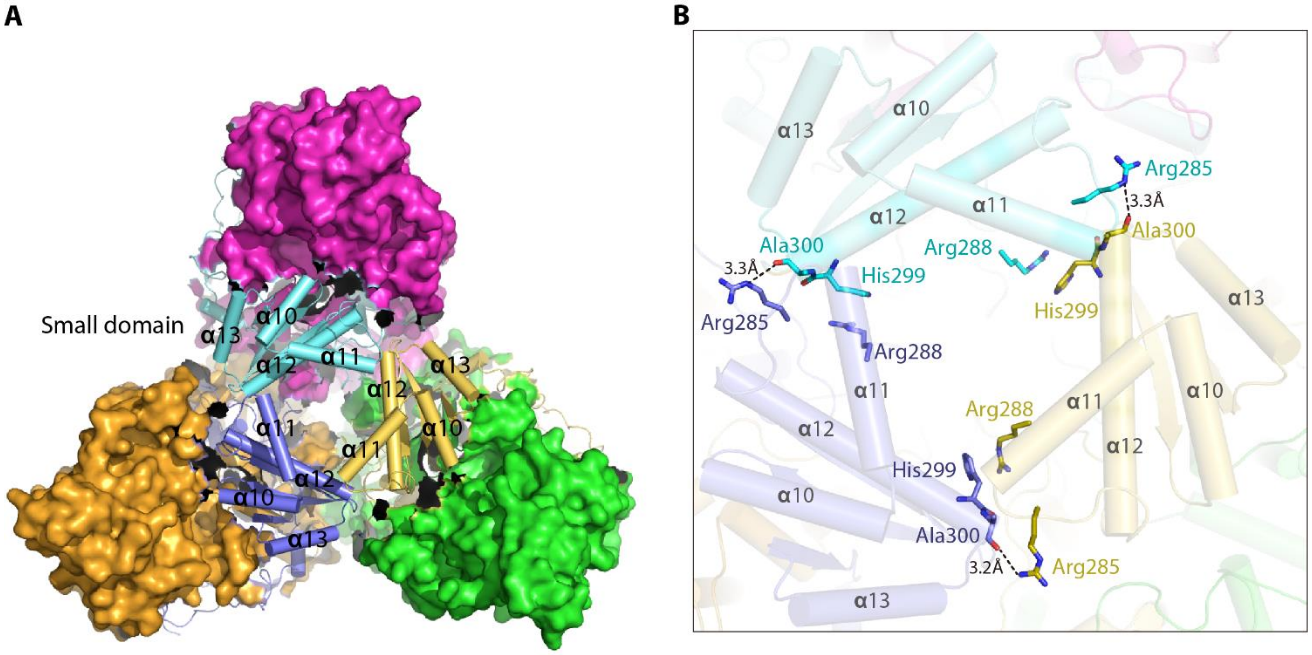


**Supplementary Figure 3.** Overall structure of the hexamer formed by crystal packing. **(A)** The small domains of three dimers are closely associated with each other at α11 and α12 helices to form a hexamer. **(B)** The hydrogen bonding interactions at the interface of the three dimers. The secondary structures from each subunit are shown in ribbon, the key amino acid residues essential for mediating

the interactions are shown in stick models, and the weak hydrogen bonding interactions are shown as dashed lines with distances labeled.

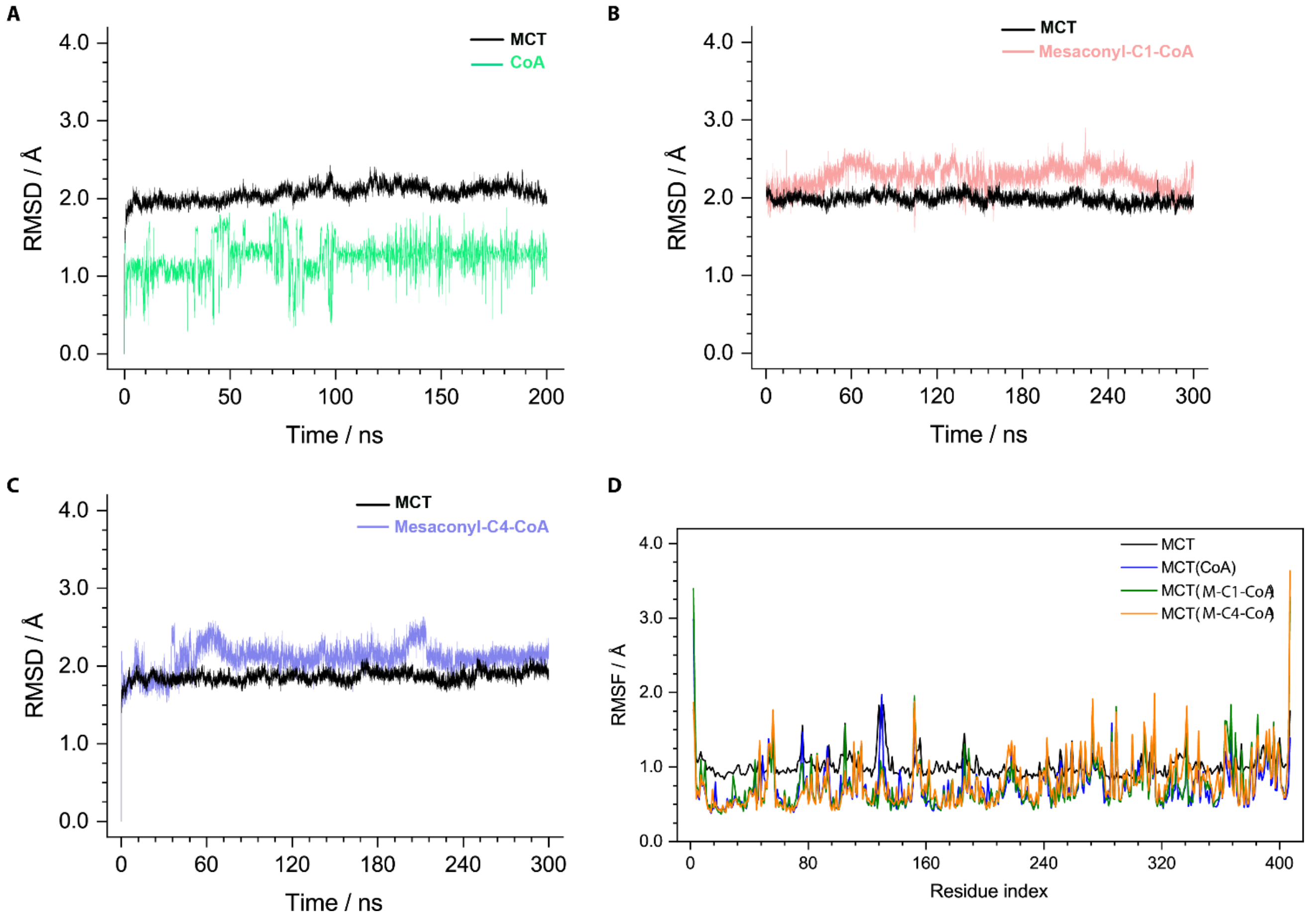


**Supplementary Figure 4.** Root-mean-square deviations (RMSDs) and root-mean-square fluctuation (RMSF) profiles of MCT and bound CoA analogs during MD simulations. **(A, B, C)** Root-mean-square deviations (RMSDs) of MCT and the bound CoA **(A)**, mesaconyl-C1-CoA **(B)** and mesaconyl-C4-CoA **(C)** during MD simulations. The curves corresponding to the RMSDs of MCT are shown in black, and the curves corresponding to the RMSDs of CoA, mesaconyl-C1-CoA and mesaconyl-C4-CoA are shown in green, salmon and blue, respectively. **(E)** Comparisons of the root-mean-square fluctuation (RMSF) profiles of the apo-MCT (black) with that of the binding structures in complex with CoA (blue), mesaconyl-C1-CoA (green) and mesaconyl-C4-CoA (orange).

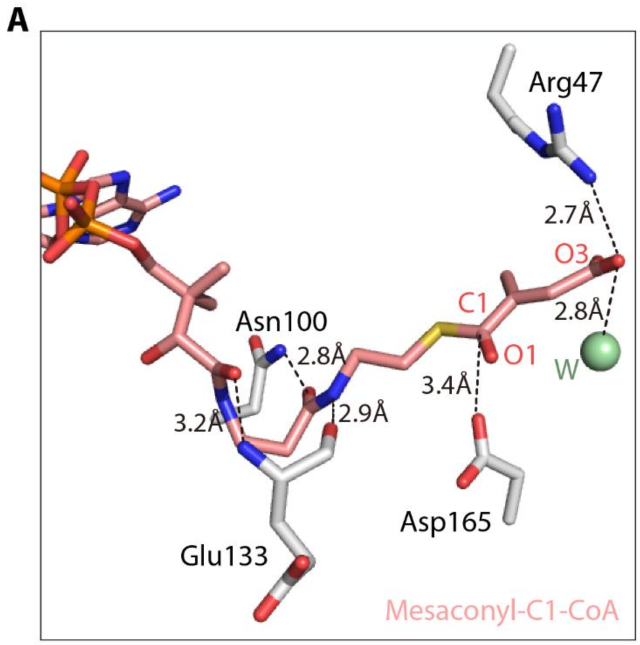


**Supplementary Figure 5.** The binding conformations of the mesaconic tail of mesaconyl-C1-CoA **(A**, salmon**)** and mesaconyl-C4-CoA **(B**, blue**)** in the substrate binding pocket of MCT. The key amino acid residues essential for accommodating the CoA analogs are shown in stick models. The hydrogen bonding and electrostatic interactions are shown as dashed lines and with the distances labeled, the water molecules are shown as green spheres (W). The carbon and oxygen atoms in mesaconyl-C1-CoA and mesaconyl-C4-CoA are labeled independently.

**Supplementary Table 1.** PISA analyses of the hexamer and dimers observed in the crystal of MCT.

| Subunit 1 | Surface (Å$^2$) | Subunit 2 | Surface (Å$^2$) | Interface area (Å$^2$) | $\Delta G^{int}$ (kcal mol$^{-1}$) | $N_{HB}$ | $N_{SB}$ | CSS |
|---|---|---|---|---|---|---|---|---|
| D | 21044 | C | 21104 | 6688.6 | -93.2 | 77 | 45 | 1.000 |
| F | 21125 | E | 21415 | 6670.3 | -94.1 | 75 | 43 | 1.000 |
| B | 21083 | A | 21266 | 6668.7 | -94.7 | 70 | 40 | 1.000 |
| | | | Average | 6675.8 | -94.0 | 74 | 43 | 1.000 |
| E | 21415 | D | 21044 | 392.1 | -3.1 | 0 | 0 | 0.044 |
| C | 21104 | B | 21083 | 391.6 | -2.8 | 0 | 0 | 0.044 |
| F | 21125 | C | 21104 | 381.5 | -3.0 | 0 | 0 | 0.044 |
| E | 21415 | A | 21266 | 379.4 | -3.0 | 0 | 0 | 0.044 |
| D | 21044 | A | 21266 | 375.8 | -3.6 | 0 | 0 | 0.044 |
| F | 21125 | B | 21083 | 359.8 | -3.6 | 0 | 0 | 0.044 |
| | | | Average | 380.0 | -3.2 | 0 | 0 | 0.044 |
| E | 21415 | B | 21083 | 114.9 | -1.5 | 0 | 0 | 0.011 |
| F | 21125 | D | 21044 | 114.8 | -1.6 | 0 | 0 | 0.011 |
| C | 21104 | A | 21266 | 108.5 | -1.8 | 0 | 0 | 0.011 |
| | | | Average | 112.7 | -1.6 | 0 | 0 | 0.011 |
| E | 21415 | C | 21104 | 91.4 | -2.7 | 0 | 0 | 0.019 |
| F | 21125 | A | 21266 | 90.1 | -2.7 | 0 | 0 | 0.019 |
| D | 21044 | B | 21083 | 83.9 | -2.7 | 0 | 0 | 0.019 |
| | | | Average | 88.5 | -2.7 | 0 | 0 | 0.019 |
